# Enhanced bacterial cellulose production in *Komagataeibacter sucrofermentans*: impact of different PQQ-dependent dehydrogenase knockouts and ethanol supplementation

**DOI:** 10.1186/s13068-024-02482-9

**Published:** 2024-02-29

**Authors:** Pedro Montenegro-Silva, Tom Ellis, Fernando Dourado, Miguel Gama, Lucília Domingues

**Affiliations:** 1 CEB–Center of Biological Engineering, University of Minho, Braga, Portugal; 2 Centre for Synthetic Biology, Imperial College London, London, UK; 3 Department of Bioengineering, Imperial College London, London, UK; 4 LABBELS–Associate Laboratory, Braga/Guimarães, Portugal

**Keywords:** Bacterial cellulose, *Komagataeibacter*, Metabolic engineering, Acetic acid bacteria, PQQ-dependent dehydrogenases, Gluconic acid, Acetic acid

## Abstract

**Background:**

Bacterial cellulose (BC) is a biocompatible material with unique mechanical properties, thus holding a significant industrial potential. Despite many acetic acid bacteria (AAB) being BC overproducers, cost-effective production remains a challenge. The role of pyrroloquinoline quinone (PQQ)-dependent membrane dehydrogenases (mDH) is crucial in the metabolism of AAB since it links substrate incomplete oxidation in the periplasm to energy generation. Specifically, glucose oxidation to gluconic acid substantially lowers environmental pH and hinders BC production. Conversely, ethanol supplementation is known to enhance BC yields in *Komagataeibacter spp.* by promoting efficient glucose utilization.

**Results:**

*K. sucrofermentans* ATCC 700178 was engineered, knocking out the four PQQ-mDHs, to assess their impact on BC production. The strain KS003, lacking PQQ-dependent glucose dehydrogenase (PQQ-GDH), did not produce gluconic acid and exhibited a 5.77-fold increase in BC production with glucose as the sole carbon source, and a 2.26-fold increase under optimal ethanol supplementation conditions. In contrast, the strain KS004, deficient in the PQQ-dependent alcohol dehydrogenase (PQQ-ADH), showed no significant change in BC yield in the single carbon source experiment but showed a restrained benefit from ethanol supplementation.

**Conclusions:**

The results underscore the critical influence of PQQ-GDH and PQQ-ADH and clarify the effect of ethanol supplementation on BC production in *K. sucrofermentans* ATCC 700178. This study provides a foundation for further metabolic pathway optimization, emphasizing the importance of diauxic ethanol metabolism for high BC production.

## Introduction

**Fig. 1:**
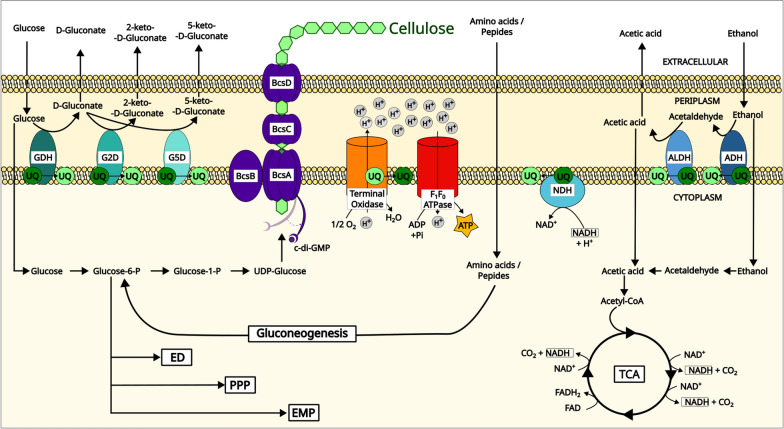
Overview of the key metabolic steps and pathways involved in BC production and energy generation in the presence of glucose and ethanol. The periplasmic oxidation of the sugars, alcohols, and sugar alcohols is linked to the electron transport chain (ETC)—the dehydrogenases transfer the reducing equivalents to ubiquinone (UQ—dark green) converting it to its reduced form, ubiquinol (UQ—light green). UQ can be further reoxidized by terminal oxidases in a process coupled with oxygen (O_2_) reduction to water (H_2_0) and the generation of a proton (H^+^) gradient. Then, F_1_F_0_ ATPase facilitates H^+^ translocation, owing to the H^+^ gradient, in a process coupled with ATP generation. In the periplasm, glucose can be partially oxidized to D-gluconate, 2-keto-D-gluconate, and 5-D-ketogluconate by PQQ-dependent glucose dehydrogenase (GDH), flavin-dependent gluconate-2-dehydrogenase (G2D), and PQQ-dependent gluconate-5-dehydrogenase (G5D). Glucose can also be imported and used for cellulose biosynthesis or metabolized through the Entner–Doudoroff (ED), Embden–Meyerhof (EMP), or Pentose Phosphate Pathway (PPP). Ethanol can be oxidized to acetaldehyde and further to acetic acid in the periplasm by PQQ-dependent alcohol dehydrogenase (ADH) and further to acetaldehyde by flavin-dependent aldehyde dehydrogenase (ALDH). When ethanol, acetaldehyde, or acetate are assimilated by the cell, the metabolites are converted to acetyl-CoA for biomass generation, or complete oxidation through the tricarboxylic acid cycle (TCA). This is also coupled with ATP generation at different points. Additionally, NADH can also be oxidized to NAD^+^ in the cytosolic membrane by NADH dehydrogenase (NADH), which is also linked to the ETC.

Bacterial cellulose (BC) is a biomaterial with properties that allow its application in a diversity of industrial sectors such as medical biotechnology, food, textiles, and electronics [1]. Acetic acid bacteria (AAB) are among the best BC producers, with *Komagataeibacter spp*. being the most remarkable within the group [2]. BC is a glucose-based polymer that grows by the sequential addition of uridine diphosphate glucose (UDP-glucose) monomers to the forming cellulosic fibril through the BC synthase complex. This is composed of 4 main sub-units—A, B, C, and D: BcsA is a glycosyl transferase whose activity is controlled by the allosteric regulator cyclic diguanylate monophosphate (c-di-GMP); BcsB is responsible for membrane anchoring, being also essential for the catalysis; while BcsC and BcsD are optional for BC biosynthesis, their presence is required for maximum productivity (Fig. 1) [3].

Efforts have been made over the years to identify conditions that allow for maximum BC productivity by *Komagataeibacter* strains, attempting to unlock the economic viability of many BC products. Such optimizations have been focused on media composition [4–8], cultural environmental conditions (pH [9, 10], temperature [11, 12], and oxygen supply [9, 13–15]), operation mode (static/agitated cultures [4, 7, 16]), and bioreactor design [9, 13–15]. Additionally, some *Komagataeibacter* strains have been engineered with some degree of success at increasing productivity [17–23]. Despite several strategies that have demonstrated significant increases, the production process is not yet cost-effective for most applications, thus imposing an entry barrier to the markets [24].

While AAB can catabolize sugars and/or alcohols in the cytosol, the group typically prefers to perform incomplete oxidation of those substrates in the periplasm, releasing the products to the culture medium [25]. This process allows AAB to control the environmental pH while rapidly generating large amounts of energy, and converting ubiquitous carbon sources into ones that most organisms cannot consume [26]. The periplasmic oxidation is usually catalysed by co-factor-dependent membrane dehydrogenases (mDHs) that transfer the electrons from substrate oxidation to the ubiquinone (UQ) pool and further to the electron transport chain (ETC) [25]. *Komagataeibacter spp.* oxidize a significant fraction of the available glucose into gluconic acid through this pathway by the action of PQQ-dependent glucose dehydrogenase (PQQ-GDH), diverting a great amount of carbon away from cellulose production (Fig. 1) [22, 27, 28]. Additionally, the consequent pH decrease negatively affects BC production [9, 10]. Two different studies report noticeable increases in BC yields in *K. xylinus* BCRC12334 [18] and *K. xylinus* BPR2001 [22], through the knockout of the *gcd* gene, which codes for PQQ-GDH. Eliminating PQQ-GDH activity in *K. xylinus* BCRC12334 directly resulted in 40% and 230% increases in BC production in static and shaken cultures, respectively [18]. On the other hand, a similar strategy was employed to engineer *K. xylinus* BPR2001, but the mutant showed impaired glucose consumption and required a lower initial pH to produce comparable amounts of BC to the control strain. A mutant that overexpresses the *glf* gene from *Z. mobilis* partially restored the ability to consume glucose with a large improvement in the conversion yield. The highest yield was reported for a strain that simultaneously overexpresses *glf* and endogenous glucokinase [22].

Ethanol supplementation is known to stimulate BC biosynthesis in *Komagataeibacter* [28–32]. Since the ethanol partial oxidation in the periplasm by the membrane-anchored PQQ-dependent alcohol dehydrogenase (PQQ-ADH) is linked to the ETC, it directly contributes to ATP generation [25] (Fig. 1). BC biosynthesis is speculated to be favoured when high intracellular concentrations of ATP are available in the cytosol. One explanation is that high ATP levels inhibit glucose-6-phosphate dehydrogenase, blocking the metabolic flow towards the pentose phosphate pathway (PPP) [32]. Additionally, ethanol supplementation has been correlated with the induction of the expression of glucokinase and genes related to the synthesis of UDP-glucose, and repression of genes involved in glycolysis and acetan formation [28]. Overall, it seems that glucose metabolism is directed towards BC biosynthesis, while the cell uses ethanol and acetate for carbon assimilation and energy generation [33].

The genome of *K. sucrofermentans* ATCC700178 codes four PQQ-dependent mDHs (PQQ-mDHs), one belonging to the methanol/ethanol family (PYD81037), and three to the glucose/quinate/shikimate family (PYD80650, PYD79881, PYD78835). In this study, we evaluate the effects of knocking out each of the four PQQ-mDHs in the genome and assess the effect on glucose conversion to gluconic acid and BC biosynthesis. Since PQQ-mDHs link directly to the ETC, we evaluate the effect of ethanol supplementation on the best BC-yielding strain, deficient in the PQQ-GDH (PYD79881), and on the mutant lacking the PQQ-ADH (PYD81037).

## Materials and methods

### Bacterial strains and growth conditions

*Komagataeibacter sucrofermentans* ATCC700178 (KS001) and derived mutants (KS002, KS003, KS004, and KS005) were propagated in Hestrin and Schramm (HS) medium [34]. Excluding carbon sources, the medium comprises 5.0 g/L yeast extract, 2.7 g/L sodium biphosphate basic, 1.15 g/L citric acid, and 0.5 g/L magnesium sulphate. The pH was set to 5.5 with 5 M hydrochloric acid if necessary. Bacterial stocks consisted of liquid culture suspensions in 15% (*v*/*v*) glycerol and were stored at – 80 °C. Before all experiments, propagation and culture synchronization were performed as follows: 5.0 mL of HS medium with 20.0 g/L glucose, 1.5% (*v*/*v*) ethanol, and 1.0% cellulase was inoculated with the strains’ glycerol stocks; the cultures were incubated for 3 days at 30 °C and 200 rpm orbital shaking, transferred to 50.0 mL of medium with the same composition, and incubated for 1 additional day in the same conditions. The bacterial biomass was then washed twice by centrifuging at 3200 *g* for 12 min and resuspended in the same volume of HS medium without a carbon source. Fermentations were then performed under different experimental conditions, being inoculated with a 1:10 volumetric ratio.

For growth curve monitoring, all bacterial strains were grown at 30 °C and 200 rpm orbital shaking in 50.0 mL HS medium with 1% (*v*/*v*) cellulase, and 20.0 g/L glucose or 1.5% (*v*/*v*) ethanol. BC production from a single carbon source was accessed by growing the strains statically at 30 °C in 20.0 mL of the same media without the addition of cellulase.

The ethanol supplementation experiment was performed by growing the bacterial strains statically at 30 °C, in 5.0 mL of HS medium with 20.0 g/L glucose and varying ethanol concentrations (0.5–5.0% (*v*/*v*)), in 25.0 mL culture tubes.

### Gene disruption of PQQ-mDHs

Genomic DNA (gDNA) extraction from KS001 was performed as described elsewhere [35]. The strains KS002, KS003, KS004, and KS005 were constructed by genomic interruption of the CRF77_01730, CRF77_05025, CRF77_00430, and CRF77_09325 *loci*, respectively, by homologous recombination with the suicide vectors KS002-KO, KS003-KO, KS004-KO, and KS005-KO.

The vectors were assembled, using the InFusion Cloning® HD kit, from gel-extracted PCR fragments. The PCR reactions were set up according to the kit’s instructions and the PCR products were purified with the QIAquick® Gel Extraction Kit. The assembly modules, for all constructs, consisted of a pUC19 backbone amplified from KTK_354 [36], a chloramphenicol resistance cassette amplified from KTK_137 [36], and upstream and downstream homologous regions, which were amplified from KS001 gDNA with sizes varying between 600 and 678 bp. The assembly reactions were transformed in *Escherichia coli* NZY5α competent cells by heat shock according to the suppliers’ protocol and transformants were selected in Luria–Bertani agar plates with 100 µg/mL ampicillin and 34 µg/mL chloramphenicol. Positive transformants were screened with colony PCR using the NZYTaq II 2 × Green Master Mix, and Sanger Sequencing. Plasmid DNA was extracted using the GenElute^™^ Plasmid Miniprep Kit. All the primers used are listed in Table 1.

**Table 1:** List of primers used for construction of suicide plasmids for knockout of the genes coding for PQQ-mDHs

Primer name	Template	Target locus	Primer sequence (5'—3')	Reference
CmR_FW	KTK_137	Chloramphenicol resistance cassette	ttgatcgggcacgtaTGATCGGCACGTAAGAGGTTCCAAC	This work
CmR_RV	KTK_137	Chloramphenicol resistance cassette	accaataaaaaacgcAGCGGAAAAGGACAAAAGTCA	This work
pUC_FW	KTK_354	Plasmid backbone (pUC19-based)	catgtgagcaaaaggccagcaa	This work
pUC_RV	KTK_354	plasmid backbone (pUC19-based)	tatggtgcactctcagtacaatctgct	This work
KS003-UF	KS001 gDNA	CFR77_05025	tgagagtgcaccataATGAATAGCCTCATACGCTCG	This work
KS003-UR	KS001 gDNA	CFR77_05025	tacgtgcccgatcaaTGTCCAGGCCACCTTCAG	This work
KS003-DF	KS001 gDNA	CFR77_05025	gcgttttttattggtGCAATCTCGGCATGTTCGAA	This work
KS003-DR	KS001 gDNA	CFR77_05025	ccttttgctcacatgTCAGTTCCCGTCAGGCAGG	This work
KS002-UF	KS001 gDNA	CFR77_01730	tgagagtgcaccataATGCGAGAAACCACCAAGAGG	This work
KS002-UR	KS001 gDNA	CFR77_01730	tacgtgcccgatcaaGACTTCAAGGTTGAACTCATGACCC	This work
KS002-DF	KS001 gDNA	CFR77_01730	gcgttttttattggtTCATGCTGGCCAATGCGAG	This work
KS002-DR	KS001 gDNA	CFR77_01730	ccttttgctcacatgTTATTGGGCTGCATTGCCTGCC	This work
KS004-UF	KS001 gDNA	CFR77_00430	tgagagtgcaccataATGATTTCTGCCGTTTTCGGAAAAAGACG	This work
KS004-UR	KS001 gDNA	CFR77_00430	tacgtgcccgatcaaGCCATCAACCGTGTAGGAACGC	This work
KS004-DF	KS001 gDNA	CFR77_00430	gcgttttttattggtGTCTGGCCAACGGCGAAT	This work
KS004-DR	KS001 gDNA	CFR77_00430	ccttttgctcacatgTTATGGCTGCTGCTCGGGAATACCG	This work
KS005-UF	KS001 gDNA	CFR77_09325	tgagagtgcaccataATGCTGCGCACTCTGCTG	This work
KS005-UR	KS001 gDNA	CFR77_09325	tacgtgcccgatcaaGGAAGTGTAATACACCACCGCCTTACAC	This work
KS005-DF	KS001 gDNA	CFR77_09325	gcgttttttattggtCGTATGACGAAAAGACCGGTATCCTGA	This work
KS005-DR	KS001 gDNA	CFR77_09325	ccttttgctcacatgTCAGTTCACGTCGTCCAGCGC	This work

Overhangs for assembly with InFusion® Cloning HD are represented in lower case letters, while template specific bases are in upper case letters

For genomic interruption of the target genes, the strain KS001 was transformed with 1 µg of suicide plasmid DNA by electroporation as described elsewhere [37]. Transformants were selected in HS-agar plates with 20.0 g/L glucose and 340 µg/mL chloramphenicol and screened with colony PCR.

### Analytical procedures

Growth curve monitoring was performed by measuring the OD_600nm_, with undiluted samples, using the Cytation™ 3 (Biotek) microplate reader. pH was qualitatively measured using pH strips. Metabolite concentrations were determined by High-Pressure Liquid Chromatography (HPLC) using a BioRad Aminex HPC-87H column at 55 °C, with a mobile phase of 5 mM H_2_SO_4_ and a flow rate of 0.5 mL/min.

### BC preparation

The BC pellicles were treated with 1% (*w*/*v*) NaOH until white or transparent and then washed with distilled water until pH neutralization. The treated membranes were then dried at 100 °C and weighed.

### Statistics and data analysis

Data treatment and pairwise *t*-tests for statistical significance were performed using the pandas and matplotlib Python libraries [38, 39]. BC production per mol of consumed glucose (BPCG), ethanol (BPCE), and carbon (BPCC) were calculated using Eqs. 1, 2, and 3, respectively:

1 $$BPCG = \frac{BC\,\,\left( g \right)}{{\Delta glu\cos e\,\,\left( {mol} \right)}},$$

2 $$BPCE = \frac{BC\,\,\,\left( g \right)}{{\Delta ethanol\,\,\left( {mol} \right)}},$$

3 $$BPCC = \frac{BC\,\,\left( g \right)}{{\left( {\Delta glu\cos e\,\,\left( {mol} \right) \ast 6} \right) + \left( {\Delta ethanol\,\,\left( {mol} \right) \ast 2} \right)}}.$$

## Results

In this work, we investigate the effect of knocking out the PQQ-mDHs present in the genome of the wild-type *K. sucrofermentans* ATCC 700178 (KS001) on BC production. The strains KS002, KS003, KS004, and KS005 were obtained by disrupting the *loci* CFR77_01730, CFR77_05025, CFR77_00430, and CFR77_09325, respectively. According to the protein annotation, the strains KS002, KS003, and KS005 lack PQQ-mDHs belonging to the glucose/quinate/shikimate family, while KS004 lacks a PQQ-mDH from the methanol/ethanol family.

The five strains were grown in agitated culture in the presence of cellulase, and in static culture for BC pellicle formation, in HS-glucose or HS-ethanol, in a range of ethanol concentrations from 0.5% to 5.0%. In all cases, the substrate consumption, extracellular metabolite accumulation, pH, and BC production were monitored.

### CFR77_05025 and CFR77_00430 are responsible for the periplasmic oxidation of glucose and ethanol, respectively

**Fig. 2:**
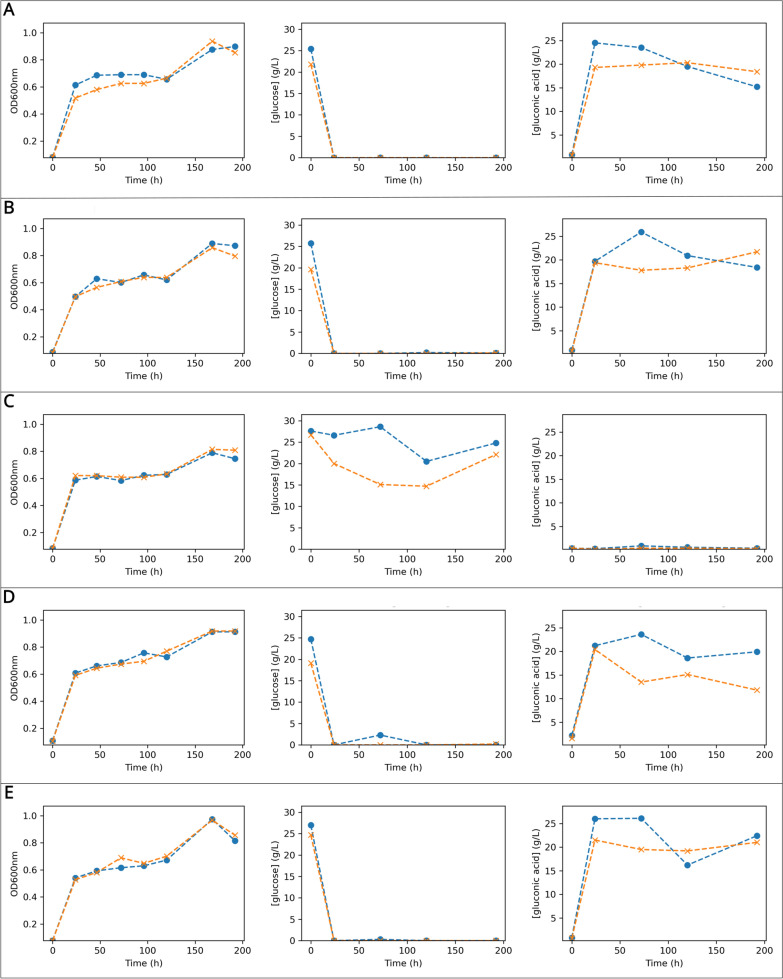
Time-course growth and metabolic profiles of agitated cultures in HS-glucose medium for five K. sucrofermentans strains—**A**: KS001; **B**: KS002; **C**: KS003; **D**: KS004; **E**: KS005. For each strain, the left panel shows OD600 nm monitoring; the middle panel depicts glucose concentration (g/L); the right panel represents gluconic acid concentration (g/L). In all panels, Replicate 1 (orange lines with dot markers) and Replicate 2 (blue lines with cross markers) are shown

**Fig. 3:**
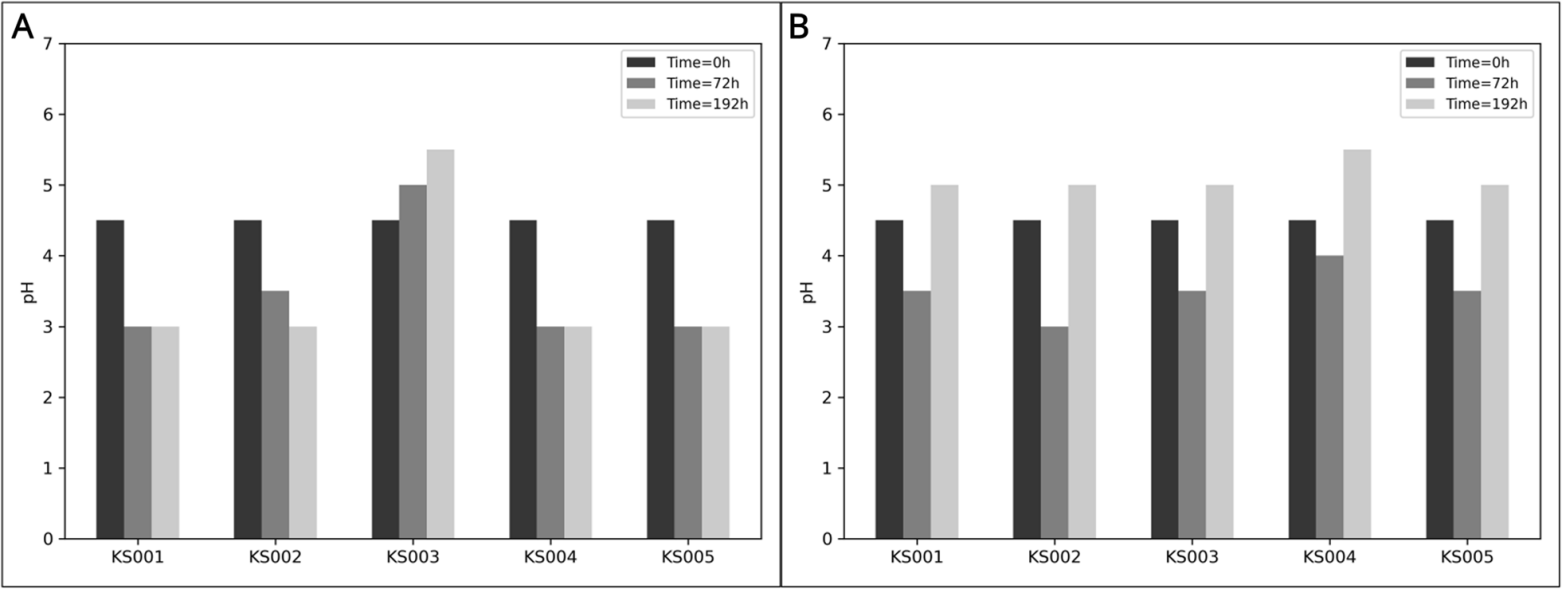
pH variation during fermentations of K. sucrofermentans strains in different media–**A**: pH measurements for strains KS001 to KS005 over time in HS-glucose medium; **B**: pH values for the same strains in HS-ethanol medium

In shake flask culture, distinct metabolic behaviours were observed among the strains. All except for KS003 were able to consume all glucose in the HS-glucose medium within the first 24 h. Over the same period, pH dropped abruptly to 3.0 due to the incomplete oxidation of glucose to gluconic acid (Figs. 2A, B, D, E, and 3A). KS003, on the other hand, slowly consumed glucose without accumulating gluconate in the culture medium. In this case, pH increased slightly until a final value of 5.5 (Figs. 2C, 3A)**.** These findings corroborate previous research [17, 18, 22], demonstrating consistent metabolic behaviours across similar studies.

**Fig. 4:**
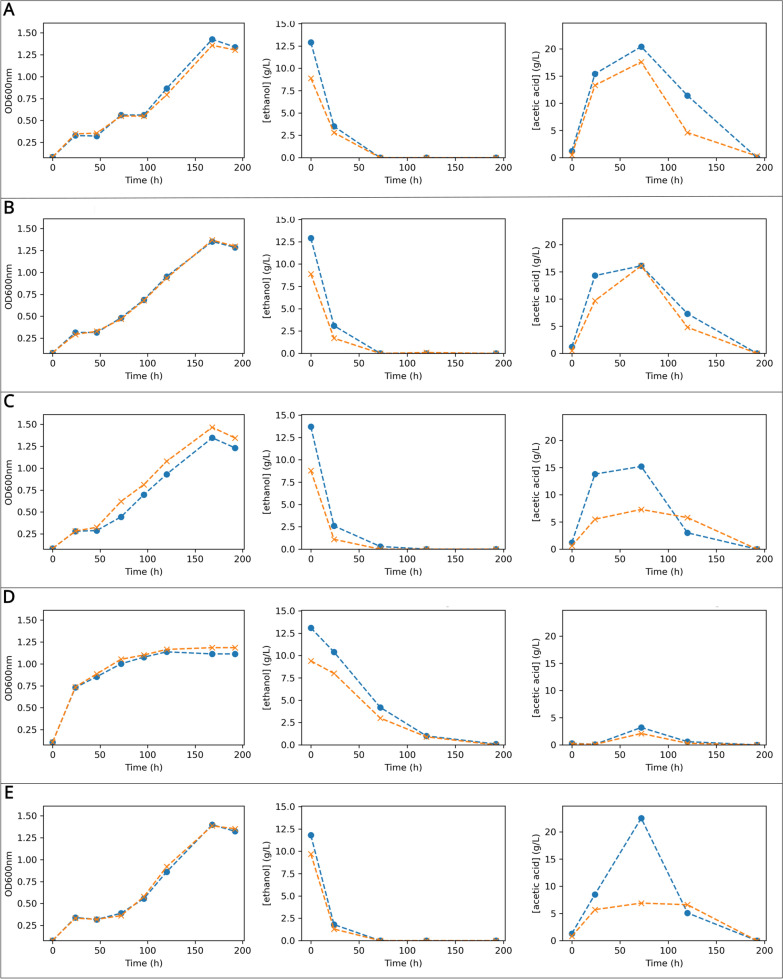
Time-course growth and metabolic profiles of agitated cultures in HS-ethanol medium for five K. sucrofermentans strains—**A**: KS001; **B**: KS002; **C**: KS003; **D**: KS004; **E**: KS005. For each strain, the left panel shows OD600 nm monitoring; the middle panel depicts ethanol concentration (g/L); the Right panel represents acetic acid concentration (g/L). In all panels, Replicate 1 (orange lines with dot markers) and Replicate 2 (blue lines with cross markers) are shown

In shake flask cultures using HS-ethanol medium, a distinct pattern in ethanol consumption and acetic acid production was observed for the strain KS004. For the remaining strains’ cultures, ethanol was depleted after 72 h of culturing in HS-ethanol, coincidently with the peak of extracellular accumulation of acetic acid (Fig. 4). However, OD_600nm_ barely varied during the second day of culturing, indicating that ethanol was probably depleted from the culture medium. After a diauxic shift that lasted for approximately 24 h, strains KS001, KS002, KS003, and KS005 started assimilating the acetate until it became undetectable by the end of the culture (Fig. 4A, B, C, E), as expected according to what is observed in other studies [28, 33, 40, 41]. KS004, on the other hand, slowly consumed the ethanol. The results suggest that ethanol was in this case being metabolized in the cytoplasm, in contrast to the remaining strains, since acetate was only detected in small amounts at the 72-h time point, coinciding with the period of steepest ethanol consumption (Fig. 4D). This result is in alignment with research in other AAB. For example, even though *Acetobacter* typically display diauxic growth in ethanol media [42], an *A. pasteurianus* SKU118 mutant deficient in the PQQ-ADH lost the ability to accumulate acetate but retained the ethanol consumption ability [43]. Regarding pH changes, all strains except KS004 displayed pH drops ranging between 1.0 and 1.5, depending on the extent of ethanol oxidation to acetate. The four cultures ended up with a final pH of 5.0 after all acetate was consumed. On the other hand, the pH in the KS004 cultures decreased slightly after 72 h due to the accumulation of small amounts of acetate in the medium and had the highest pH values at the end of the culture with a value of 5.5 (Fig. 3B).

In summary, the strains KS003 and KS004 display an absence of extracellular accumulation of large amounts of gluconic and acetic acids, respectively. This strongly suggests that the PQQ-mDHs coded by the *loci* CFR77_05025 and CFR77_00430 are responsible for the periplasmic partial oxidation of glucose and ethanol, respectively.

### The strain KS003 shows improved BC production in static culture

The four knockout mutants were further compared with the wild-type strain KS001 for their ability to produce BC in static culture in the presence of glucose or ethanol as the sole carbon source.

**Fig. 5:**
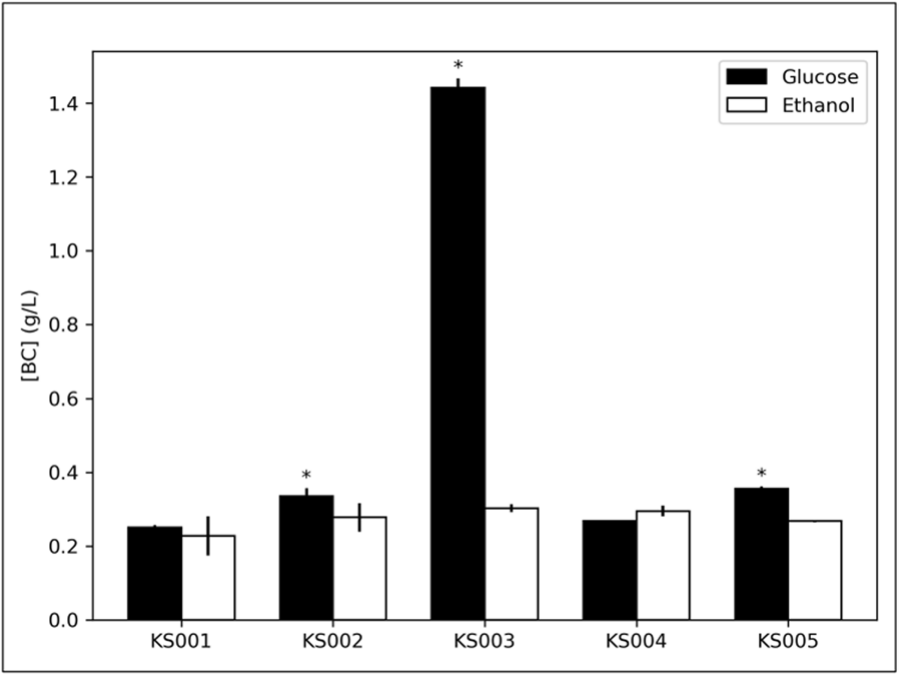
BC production (g/L) of K. sucrofermentans strains KS001 to KS005 grown in HS-glucose (black bars) and HS-ethanol (white bars) media, in static culture. Error bars denote standard deviations. * Statistically significant difference in BC concentration compared to the KS001 strain for the corresponding carbon source (*N* = 2; *p* < 0.05)

In the experiment using HS-glucose medium, all mutants achieved higher average VPE than KS001, but the improvement was not statistically significant for KS004 (*p* = 0.05). In ascending rank of BC production, KS001 (0.25 ± 0.01 g/L) < KS004 (0.27 ± 0.01 g/L) < KS002 (0.34 ± 0.02 g/L) < KS005 (0.36 ± 0.01 g/L) < KS003 (1.44 ± 0.01 g/L). Although most strains showed marginal improvement in BC production, KS003 remarkably showed a 5.77-fold increase when compared to KS001 (Fig. 5). This finding aligns with previous observations in other *Komagataeibacter* species, where a knockout of PQQ-GDH enzyme led to increased BC production in glucose media [17, 18, 22].

In HS-ethanol, all mutant strains achieved non-significantly higher VPE than KS001, ranking as follows: KS001 (0.23 ± 0.04 g/L) < KS005 (0.27 ± 0.03 g/L) < KS002 (0.28 ± 0.03 g/L) < KS004 (0.30 ± 0.01 g/L) < KS003 (0.30 ± 0.01 g/L) (Fig. 5).

### Ethanol concentration modulates BC production and metabolic responses in *Komagataeibacter* strains

It is widely reported in the literature that ethanol supplementation drives glucose towards BC production in *Komagataeibacter spp.*, boosting the yield of the polymer [17, 28, 30, 32, 44]. As AAB partially oxidize glucose and ethanol in the periplasm while supplying the reducing equivalents to the ETC, both reactions are directly coupled with energy generation [25]. Since KS003 and KS004 lack PQQ-GDH and PQQ-ADH, respectively, we decided to evaluate the impact of knocking out those genes on the typical BC production boost observed in cultures where both glucose and ethanol are present.

**Fig. 6:**
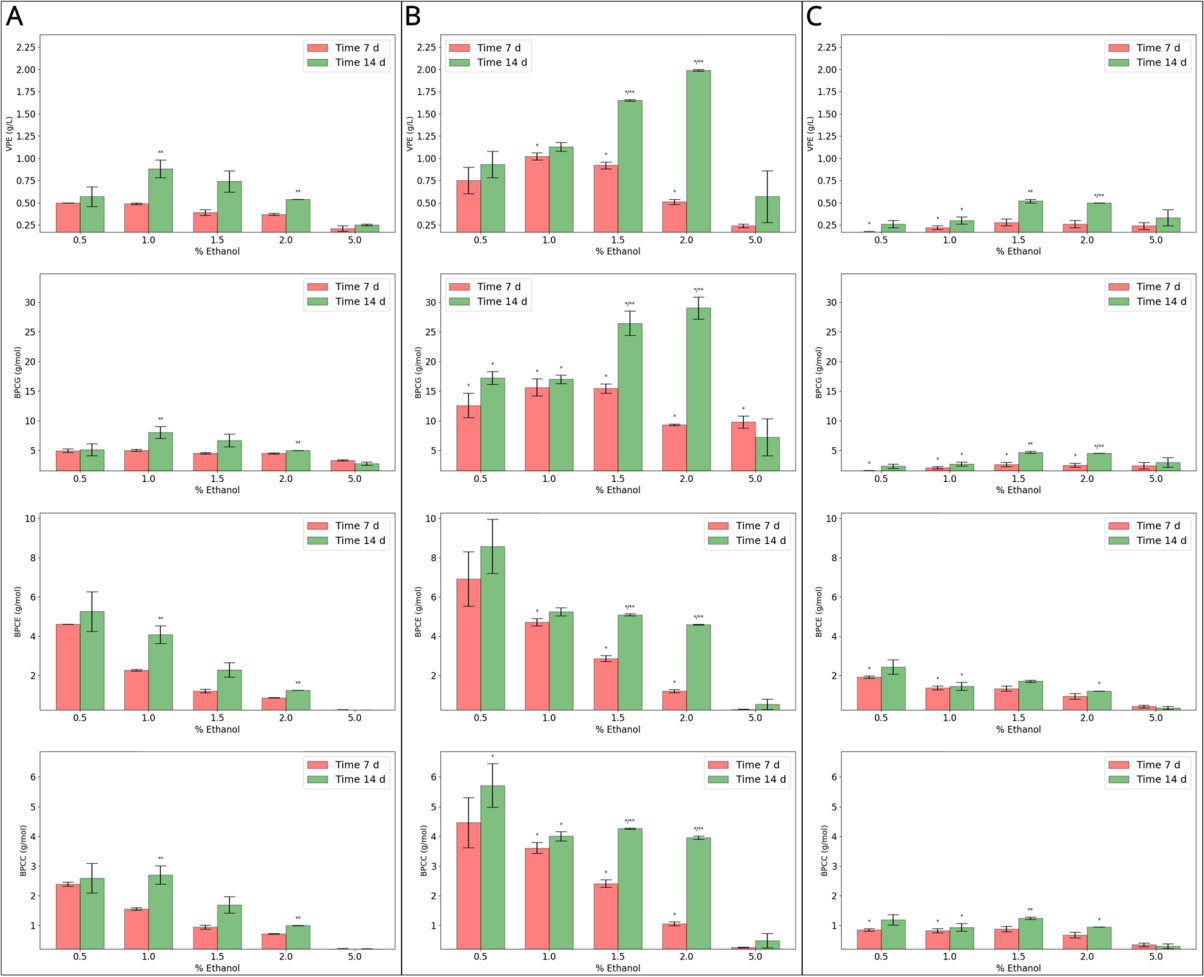
Comparative analysis of bacterial cellulose production efficiency in K. sucrofermentans strains in HS-glucose-ethanol media in a range of ethanol concentrations, in static culture—**A**: KS001; **B**: KS003; **C**: KS004. The top panel (VPE) represents volumetric production efficiency (g/L), the second panel (BPCG) shows bacterial cellulose production per consumed glucose (g/mol), the third panel (BPCE) denotes bacterial cellulose production per consumed ethanol (g/mol), and the bottom panel (BPCC) illustrates bacterial cellulose production per consumed carbon (g/mol). Red bars represent data at 7 days, while green bars denote data at 14 days. Error bars indicate the standard deviation. * Significance against strain KS001 for the same condition and time point (*N *= 2; *p* < 0.1). ** Significance when comparing 14 days to 7 days for a given condition (*N* = 2; *p* < 0.1)

By testing a range of increasing concentrations, we were able to observe that the optimal ethanol levels required for peak BC production in the strains KS003 and KS004 shifted when compared to KS001. Specifically, while KS001 has its peak of BC production at an ethanol concentration of 1.0% (*v*/*v*), KS004 and KS003 had their maximum VPE at 1.5% (*v*/*v*) and 2.0% (*v*/*v*), respectively. The shift in the optimal ethanol concentration might reflect a lower competition for ubiquinone in the ETC, which is a typical limiting step in catalysis by PQQ-mDHs [25]. The VPE at each strain’s optimal ethanol concentration ranked as follows: KS004 (0.52 ± 0.02 g/L) < KS001 (0.88 ± 0.1 g/L) < KS003 (1.99 ± 0.01 g/L) (Fig. 6A, B, C—top panels).

Knocking out the PQQ-ADH (strain KS004) was not favourable for BC production when glucose and ethanol were simultaneously present in the culture medium (Fig. 6C). On the other hand, the absence of periplasmic glucose dehydrogenase activity was beneficial since KS003 had an increase of 2.26-fold in BC production when compared to KS001 at their respective optimal ethanol concentrations (Fig. 6B). While a mutant lacking the PQQ-GDH was previously found to benefit from ethanol supplementation, this is the first report of a systematic comparison of the response to different ethanol concentrations against the wild-type strain [17].

**Fig. 7:**
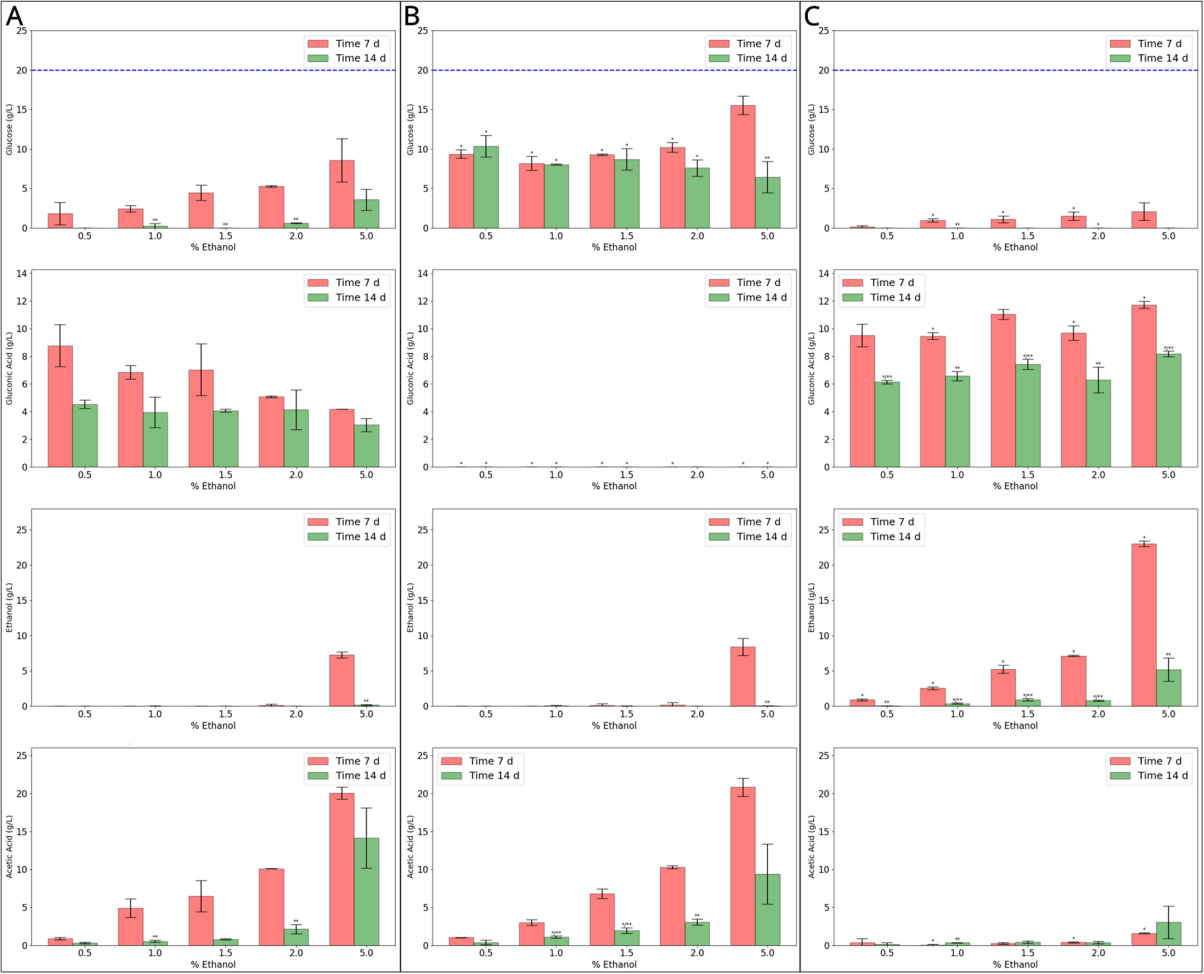
Metabolic profiles of K. sucrofermentans strains in HS-glucose-ethanol media. Each panel shows the residual concentration of (from top to bottom) glucose, gluconic acid, ethanol, and acetic acid (all in g/L) for strains KS001 **A**, KS003 **B**, and KS004 **C**. Red and green bars indicate measurements at 7 and 14 days, respectively; error bars represent standard deviation; the horizontal blue dashed line represents the initial glucose concentration (g/L). * Significant differences from strain KS001 under identical conditions (*N* = 2; *p *< 0.1). ** Significant differences between the time points for each strain (*N* = 2; *p* < 0.1)

The initial 7 day cultivation period revealed a negative correlation between escalating ethanol concentrations and BC production for KS001 (Fig. 6A—top panel). This lagging effect was manifested through heightened levels of residual glucose present in the medium, coupled with a diminished formation of gluconate (Fig. 7A). Slower glucose consumption and gluconate production rates in dual carbon source media, in comparison with media with glucose as the sole carbon source, were expected [17]. Moreover, a relationship was observed between acetate accumulation and the initial concentration of ethanol. After 7 days, ethanol was scarcely detected in the cultures of KS001 except in those initiated with a 5.0% (*v/v*) ethanol concentration. Transitioning into the subsequent week of cultivation, a shift in the metabolic dynamics was noted. Glucose was predominantly detected in cultures supplemented with 2.0% and 5.0% ethanol, while a standardization occurred in gluconate concentrations, stabilizing at approximately 4 g/L across all experimental conditions (Fig. 7A). At first sight, this result contrasts with what was observed by Ryngajłło et al., where the supplemented cultures left a substantial amount of residual glucose and produced less gluconate. However, in contrast to our results, there was still a large amount of acetate in the medium, and it is unclear whether glucose consumption and gluconate production would have further progressed [28]. In terms of BC production, a substantial increase was measured, particularly in the conditions with ethanol concentrations calibrated to 1.0% (*v*/*v*) and 1.5% (*v*/*v*). In this period an acceleration in BC production is apparent, coincidently with the assimilation of acetate, but this increase in respect to the first week was only significant (*p* < 0.1) for the cultures initiated with 1.0% and 2.0% (*v*/*v*) due to the variability between the replicates (Figs. 6A and 7A). A substantial increase in BC production during the acetate assimilation phase was also observed by Yunoki et al. in *K. xylinus* ATCC 10245 in a similar culture medium composition [33].

Regarding strain KS003, a distinct metabolic behaviour was observed when contrasted with KS001, characterized by the absence of gluconate formation. This phenomenon is closely tied to the pronounced retention of glucose in the medium, with 30–50% remaining post-experiment. Despite this, the KS003 strain maintained residual ethanol and acetate levels comparable to those found in KS001 at both examined time points (Fig. 7B). When focusing on BC production, the initial week exhibited a minor difference between the conditions with initial ethanol concentrations of 0.5% (*v*/*v*) and 1.0% (*v*/*v*), while higher values resulted in lagged exopolysaccharide secretion. This trend sharply contrasted in the second week where acetate assimilation spurred significant boosts in BC production (*p* < 0.1), when compared to the first week, in the cultures initiated with ethanol concentrations of 1.5% (*v*/*v*) and 2.0% (v/v) (Fig. 6B). The increase in these two conditions, in comparison with KS001, was also significant (*p* < 0.1).

A distinct pattern of BC production was observed for the strain KS004. Despite a significant 1.9-fold augmentation from the first to the second week of cultivation (*p* < 0.1) at the ethanol concentration of 2.0% (Fig. 6C), it is critical to note the strain's overall diminished BC production. This observation starkly contrasts with its ability to maintain the lowest levels of residual glucose, and higher conversion to gluconate, across all examined conditions and temporal checkpoints (all significant; *p* < 0.1). Furthermore, the KS004 strain demonstrated a restrained extracellular acetate accumulation, maintaining low levels in most conditions with a notable increase observed in cultures supplemented with a 5.0% ethanol concentration (Fig. 7C).

The BPCG in the KS001 strain tended to decrease with increased initial ethanol concentrations during the first week of cultivation. However, with extended culturing to 14 days, a substantial increase in BPCG was observed in cultures with 1.0% (*v*/*v*) and 1.5% (*v*/*v*) ethanol concentrations. The 1.0% (v/v) ethanol condition reached the highest BPCG of 8.03 ± 1.01 g/mol for this strain (Fig. 6A—second panel). The BPCE showed a consistent decline with increasing ethanol concentrations across both the 7- and 14-day culturing periods (Fig. 6A—third panel). In the first 7 days, there was a downward trend in BPCC as ethanol concentration increased. In the second week, significant increases in BPCC were noted for the 1.0% and 2.0% ethanol conditions (*p* < 0.1). The BPCC was notably higher at lower ethanol concentrations (0.5% and 1.0% *v*/*v*) (Fig. 6A – bottom panel).

During the initial cultivation week, strain KS003 demonstrated a declining BPCG with increased ethanol concentration. However, a significant increase in BPCG was observed in the 1.5% (*v*/*v*) and 2.0% (*v*/*v*) ethanol conditions over 14 days (*p* < 0.1), with increases of approximately 1.71- and 2.1-fold, respectively. The optimal condition of 2.0% (*v*/*v*) ethanol yielded an average BPCG of 29.04 ± 1.87 g/mol, which was the highest value in the experiment (Fig. 6B—second panel). In contrast to KS001, KS003 showed a more stable BPCE in the second week of culturing, maintaining levels around 4.97 ± 0.28 g/mol across mediums with 1.0%, 1.5%, and 2.0% (*v*/*v*) ethanol. In media with 1.5% (*v*/*v*) and 2.0% (*v*/*v*), at the end of the experiment, the increase in BPCE was significantly higher when compared with the strain KS001 and with the intermediate time point (*p* < 0.1) (Fig. 6B – third panel). The BPCC pattern in KS003 over the 2 week period showed the highest yields at the lowest tested ethanol concentration. The BPCC levelled off at an average of 1.58 ± 0.03 g/mol in trials with 1.5% and 2.0% initial ethanol concentrations, with a marked downturn at 5.0% (*v*/*v*) ethanol concentration (Fig. 6B—bottom panel).

Strain KS004 exhibited the lowest BPCGs of the experiment. The highest BPCG was 4.68 ± 0.18 g/mol under the 1.5% (*v*/*v*) ethanol condition, which was considerably lower when compared to the other two strains (Fig. 6C–second panel). Throughout the initial 7 day period, the BPCC in KS004 was slightly affected within the ethanol range of 0.5–2.0% (*v*/*v*), with an average value of 0.23 ± 0.02 g/mol. However, a more fluctuating BPCC pattern was observed over 14 days, with values oscillating between 0.30 ± 0.05 and 0.41 ± 0.01 g/mol (Fig. 6C—bottom panel). KS004 demonstrated restrained benefits from ethanol supplementation due to the removal of PQQ-ADH, contrasting with other strains that showed improved BC production in ethanol-containing media.

## Discussion

Ethanol supplementation in glucose-containing media is an established method to enhance BC production [28, 30, 33, 41]. *Komagataeibacter spp.* express mDHs specific for the oxidation of both substrates in a reaction coupled with energy generation by electron channelling through the ETC [25, 28]. Interestingly, multiple studies reported that the attenuation, or elimination, of extracellular accumulation of gluconic acid is typical in high BC-producing strains [17, 18, 22]. As PQQ-mDHs are key players in the metabolic landscape of AAB [22], combining both strategies can generate useful insights for a better understanding of their role in BC production and allow the achievement of better yields.

Among the mutants generated in this work, the strains KS003 and KS004 stood out due to their inability to accumulate gluconate and acetate in the medium, respectively. This indicates that the loci CFR77_05025 and CFR77_00430 code the PQQ-mDHs responsible for the periplasmic oxidation of glucose and ethanol in *K. sucrofermentans*, respectively. While neither strain was able to show significantly increased BC production when grown in HS-ethanol, by comparison with KS001, KS003 showed a substantial increase in HS-glucose.

Three previous studies have reported the loss of gluconate production by interruption of the *gcd* gene, with the respective KO strains preventing the typical pH drop [17, 18, 22]. Shigematsu et. al and Kuo et. al reported slower glucose consumption rates, in *K. xylinus* BPR 2001 and *K. xylinus* BCRC 12334, respectively, with the KO strains reaching higher OD600_nm_ than the unmodified ones, in agitated cultures with cellulase [17, 18]. Without further optimization, the KO strains consumed all available glucose and achieved 1.4- and 1.7-fold increased BC production in the static mode of cultivation, respectively. Knocking out the *gcd* gene in *K. xylinus* CGMCC 2955, on the other hand, resulted in a strain with severe growth, BC production, and glucose consumption deficiencies, thus requiring further modifications to improve the yield [22]. While the overexpression of the glucose facilitator protein (*glf* from *Zymomonas mobilis*) and the native glucokinase (*glk*) did not fully restore the glucose consumption ability, it has improved its conversion to BC, thus unlocking a similar volumetric yield to the control strain from a lower glucose concentration. In our study, KS003 was able to reach the same OD_600nm_ as KS001 in an agitated culture in the presence of cellulase. However, the simultaneous production and degradation of BC caused oscillating glucose levels in the medium. This was only observed in this strain’s cultures, thus evidencing its impaired glucose consumption. As the glucose oxidation to gluconic acid occurs in the periplasm, and the latter is mostly released to the extracellular medium, the fact that the strain KS003 can reach a similar OD_600nm_ as KS001 can be an indicator that both strains are equally efficient at importing glucose to the cytoplasm. Nonetheless, the strain was able to produce 5.77-fold more BC than the unmodified strain in static cultures with glucose as the sole carbon source.

All strains showed low BC production levels with ethanol as the sole carbon source but were able to metabolize the alcohol with no exception. However, acetate was barely detected in the extracellular medium, in agitated cultures with the strain KS004. This is corroborated by the pH measurements. The residual extracellular levels of acetate are probably due to the efflux by ABC transporters, associated with the high acetic acid resistance phenotype typically observed in bacteria from the *Komagataeibacter* genus [28, 45].

In *Komagataeibacter*, ethanol metabolism has two distinct phases: (1) ethanol oxidation in the periplasm, which causes acetate to accumulate in the medium; and (2) acetic acid assimilation [28, 32, 33]. The prioritization of extracellular accumulation of acetate is corroborated by gene expression in *K. xylinus* E25, where the authors found acetate kinase, succinyl-CoA:acetate-CoA-transferase, and most genes from the tricarboxylic acid (TCA) cycle to be down-regulated while ethanol is not depleted from the culture medium [28]. Our results suggest that KS004 was able to metabolize the ethanol, but through the cytosolic route, instead of the signature periplasmic incomplete oxidation. While the growth curves in the remaining strains show the occurrence of a diauxic shift, the same was not observed in KS004 (Fig. 4D—left panel). Given that KS004 does not accumulate acetate, and that the strain displays a distinct growth curve, our results point to a different mode for ethanol metabolization than the typical two-staged metabolism. This was previously observed in a PQQ-ADH-deficient *A. pasteurianus* mutant, that ceased to accumulate acetate extracellularly while retaining the ethanol metabolization ability [43].

Bacteria from the *Komagataeibacter* genus seem to increase the glucose flow towards BC production and its necessary precursors in ethanol supplementation setups [28, 33]. The presence of ethanol induced the expression of galP3, a putative glucose transporter, glucokinase, UDP-glucose pyrophosphorylase, and phosphoglucomutase, in *K. xylinus* E25. Repression of the *gcd* gene, and others involved in glycolysis and the PPP was also observed. Overall, their results suggested enhanced direct polymerization of glucose instead of its use for biomass and energy [28]. Our results are in alignment with their observations since KS001, KS003, and KS004 showed a higher volumetric production when grown in an ethanol supplementation setup than in the single carbon source experiment.

Ethanol supplementation is usually associated with slower glucose consumption and lower gluconate production. In strain KS001, the glucose consumption and gluconate production rates decrease with the increase of the initial concentration of ethanol during the first week of cultivation, which is when the ethanol oxidation phase occurs. However, strain KS004 was significantly faster at consuming the glucose and produced more gluconate than strain KS001. As our results suggest that the strain directly oxidizes ethanol to CO_2_ and H_2_0 in the cytoplasm, thus not requiring a diauxic shift to allow acetate assimilation [43], it is then possible that KS004 does not operate under the same regulatory rules.

During the ethanol oxidation phase, the ETC is fed electrons mainly from the PQQ-mDHs, since the NADH dehydrogenase is down-regulated [25, 28]. However, in *K. xylinus* E25 the ubiquinol reoxidation to UQ appeared to be on demand since up-regulation of the cytochrome ba_3_ was observed, thus suggesting a high respiration rate during ethanol oxidation [28]. Since the substrate oxidation reactions of PQQ-GDH and PQQ-ADH both require the reoxidation of PQQ, an increase in the activity of the former might be partially caused by a decreased competition for UQ in the strain KS004, due to the absence of the latter [25]. This might also explain why both KS003 and KS004 show a preference for higher concentrations of ethanol than KS001. Nonetheless, our results suggest that the absence of PQQ-ADH is, at least partially, being compensated by higher PQQ-GDH activity.

After 7 days, glucose concentrations between 8.19 ± 0.64 g/L and 10.19 ± 0.43 g/L were measured in cultures of the strain KS003 with up to 2.0% (*v*/*v*) ethanol. Those values were all significantly superior to ones obtained with the strain KS001 under similar conditions, which further corroborates that strains lacking a functional PQQ-GDH show slower glucose consumption [17, 18, 22]. Additionally, a putatively increased expression of a galP3 homolog in the presence of ethanol, as observed by Ryngajłło et al. [28], did not fully restore the ability of KS003 to import glucose. Despite that, the strain KS003 showed significantly higher VPEs than KS001 for the conditions with 1.0–2.0% ethanol. In fact, during the first half of the culturing period, the strain KS003 was able to produce more BC than KS001 during the full duration of the experiment.

On one hand, while ethanol supplementation narrowed the fold increase between KS003 and KS001 in terms of BC production, by comparison with cultures in HS-glucose, the strain lacking the PQQ-GDH was still the best-performing strain in the experiment. On the other hand, KS004 was the worst BC producer in the presence of both carbon sources. As previously mentioned, substrate oxidation by PQQ-mDHs is tightly linked to the ETC and thus ATP biosynthesis [25]. As BC production is an energy-demanding process, the extra ATP generated by ethanol oxidation is thought as one of the factors that cause increased yields [28, 30, 32, 33, 41]. Additionally, ATP can have a regulatory effect by inhibiting the activities of glucose-6-phosphate dehydrogenases [32].

Two different studies that compare the intracellular ATP levels in glucose-based media, with and without ethanol supplementation, obtain contrasting results. While Naritomi et al. verified that ethanol supplementation resulted in increased ATP levels in *Komagataeibacter sucrofermentans* BPR 3001A [32], Ryngajłło verified the opposite in *K. xylinus* E25 [28]. The striking differences between both studies were the sugars used and the fermentation mode. Naritomi et al. grew the bacteria in continuous culture, with fructose and ethanol feed. Ryngajłło et al. performed a static batch culture where glucose was used instead of fructose. The PQQ-ADH activity is concentration dependent [46] and, due to the diauxic metabolism that *Komagataeibacter* display in the presence of ethanol, the bacteria were continuously generating ATP at a virtually fixed rate, in Naritomi et al. [32]. In batch culture, the ethanol concentration decreases over time, partially explaining why Ryngajłło et al. measured lower ATP levels at the latter stage of the ethanol oxidation, while the TCA was still down-regulated. Interestingly, the ATP levels were still much lower during the acetate assimilation phase when compared with the non-supplemented medium, which the authors justify with an increased energy demand due to efflux of acetate, nutrient import from the culture medium, and BC biosynthesis [28].

The results of the ethanol supplementation experiment show that the higher the initial concentration of ethanol is in the medium, the longer it takes for the BC production boost to occur. Although the BC production after 7 days was higher than in HS-glucose both for KS001 and KS003, a significant boost occurred during the second week of cultivation in mid-range ethanol concentrations. Although our study lacks temporal resolution, one assumes that the higher the ethanol concentration, the more prolonged the ethanol oxidation phase will be. The increased lag for the production boost to occur as the initial ethanol concentration increases suggests that the major production of BC occurs during the acetate assimilation phase, as observed in other studies [28, 33]. This was especially visible for the strain KS003 which suffered a penalizing effect with the increase from 0.5 to 2.0% ethanol during the first week but prospered in BC production over the second week in more acetic environments. Interestingly, the glucose concentration in the media barely varied within the same timeframe.

Upon ethanol depletion from the culture medium, a diauxic shift occurs and *Komagataeibacter* species start catabolizing the previously accumulated acetate. Kornmann (2003) and Sakurain (2012) reported that the TCA cycle is more active on acetate than it is on ethanol in *K. xylinus* I2281 and *A. aceti* NBRC 14818, respectively. It appears that during the second phase of acetate utilization, the acetyl-CoA synthase plays a major role in converting the acetate into acetyl-CoA, which can further be used to generate biomass or be channelled to the TCA cycle for energy generation [41, 42]. When comparing the BPCEs and BPCCs of the strains KS001 and KS003, both metrics showed an inverse relationship with the ethanol concentration during the first week, when the ethanol was converted to acetate, which was not immediately assimilated (Figs. 6A, B, 7A, B). Since acetate is assimilated upon cessation of ethanol oxidation [28, 30, 33, 41, 42], further BC production could only impact the BPCE positively. The fact that, at the end of the fermentation with the strain KS003, the BPCE barely changed within cultures initiated with 1.0–2.0% (*v*/*v*) ethanol suggests that BC production was nearly proportional to the amount of acetate that is being assimilated in such conditions.

Interestingly, the evidence in the literature suggests that carbon from acetate is unlikely to be incorporated into BC. Yunoki et al. (2004) conducted tests in *K. xylinus* with D-(1-^13^C)-, D-(2-^13^C)-, and D-(6−^13^C)-glucose, and (1−^13^C)-ethanol labelling, demonstrating that in ethanol supplementation setups with ^13^C-labelled ethanol, there is no evidence of labelled cellulose following ethanol oxidation or acetic acid assimilation [33]. That study also observed impaired BC production during the ethanol oxidation phase and a subsequent boost during acetate assimilation. The absence of labelled BC in cultures with (1−^13^C)-ethanol indicates that acetate was rather fuelling the metabolism through the TCA cycle. The rearrangement of the glucose ring in BC synthesized in cultures with differently labelled glucose suggests that not only ethanol favours direct polymerization of glucose but also gluconeogenesis from peptides and amino acids imported from the culture medium [33]. The transcriptome of *K. xylinus* E25 also suggests that the active transport of nutrients from the culture medium is increased by ethanol supplementation [28]. Moreover, we were unable to find a coding sequence for homologs of *E. coli*’s phosphoenolpyruvate carboxykinase in the genome of the large majority of *Komagataeibacter spp.* through a BLAST search (not shown). This might be indicative that a metabolic route that could divert oxaloacetate to gluconeogenesis might be absent. Additionally, independent studies have found that supplementation with glucogenic and amphibolic amino acids boosts BC production in *Komagataeibacter spp* [47, 48]. One study reports that L-methionine (glucogenic) boosts BC production in 50%, in basal medium, in *Komagataeibacter sucrofermentans* BPR2001 [47]. Another study reports that aspartic acid and serine (glucogenic), and phenylalanine (amphibolic) boost BC production in *Komagataeibacter intermedius* V-05 [48].

Altogether, the evidence in the literature suggests that ethanol promotes a more efficient use of glucose and nutrients contained in the peptone and yeast extract, while not being a source of carbon for BC production [28, 33, 47, 48]. This would imply that, if that is the case, most of the glucose monomers in the BC being produced by the strain KS003 during the acetate assimilation phase were originated by neogenesis from imported amino acids, since the levels of glucose barely change during that period. This work suggests that the PQQ-ADH is an important component at increasing the BC productivity in the presence of ethanol. PQQ-ADH overexpression in KS003 might further improve the strain’s productivity by increasing the rate of ethanol oxidation while simultaneously conferring acetic acid tolerance [25]. This might have a positive influence on the strains’ bioenergetics and, consequently, on BC productivity.

## Conclusions

Our study underlines the importance of membrane-anchored PQQ- mDHs, specifically PQQ-GDH and PQQ-ADH, in BC production by *K. sucrofermentans*. The metabolic differences in the strains KS003 and KS004, characterized by altered patterns of gluconate and acetate extracellular accumulation, respectively, allowed a better understanding of their role in substrate oxidation and the impact on BC yield. Notably, the impaired glucose consumption coupled with enhanced BC production in KS003, despite its metabolic limitations, highlights the complex interplay between pH, metabolic fluxes, and product synthesis.

Furthermore, our findings suggest that ethanol supplementation, while might not directly contribute carbon for BC biosynthesis, plays a crucial role in modulating cellular metabolism towards optimal BC production, possibly through the regulation of energy generation and nutrient assimilation pathways. Additionally, our results suggest that the extracellular accumulation of acetate is advantageous over direct metabolization of ethanol through the cytosolic route. The importance of PQQ-ADH for increased BC yield through ethanol supplementation was already hypothesized in the literature. However, to our knowledge, this is the first report of a *Komagataeibacter* strain unable to express a functional PQQ-ADH displaying a restrained benefit from ethanol supplementation.

Therefore, this study establishes the groundwork for further strain improvement, by offering potential strategies for targeted manipulation of metabolic pathways to enhance BC production.

## Data Availability

The datasets used and/or analysed during the current study are available from the corresponding author on reasonable request.

## Abbreviations

AAB: Acetic acid bacteria
ADH: Alcohol dehydrogenase
BC: Bacterial cellulose
BPCC: Bacterial cellulose production per mol of consumed carbon
BPCE: Bacterial cellulose production per mol of consumed ethanol
BPCG: Bacterial cellulose production per mol of consumed glucose
c-di-GMP: Cyclic diguanylate monophosphate
ED: Entner–Doudoroff
ETC: Electron transport chain
EMP: Embden–Meyerhof
gDNA: Genomic DNA
HPLC: High-pressure liquid chromatography
HS: Hestrin and Schramm
mDH: Membrane dehydrogenase
PPP: Pentose phosphate pathway
PQQ: Pyrroloquinoline quinone
PQQ-ADH: Pyrroloquinoline quinone-dependent membrane alcohol dehydrogenase
PQQ-GDH: Pyrroloquinoline quinone-dependent membrane glucose dehydrogenase
PQQ-mDH: Pyrroloquinoline quinone-dependent membrane dehydrogenase
TCA: Tricarboxylic acid cycle
UDP: Uridine diphosphate
UQ: Ubiquinone
VPE: Volumetric production efficiency
